# How musical expertise shapes speech perception: evidence from auditory classification images

**DOI:** 10.1038/srep14489

**Published:** 2015-09-24

**Authors:** Léo Varnet, Tianyun Wang, Chloe Peter, Fanny Meunier, Michel Hoen

**Affiliations:** 1 Lyon Neuroscience Research Center, CNRS UMR 5292, INSERM U1028, Auditory Language Processing (ALP) research group, Lyon, France; 2 Laboratoire sur le Langage le Cerveau et la Cognition, CNRS UMR 5304, Auditory Language Processing (ALP) research group, Lyon, France; 3 Université de Lyon, Université Lyon 1, Lyon, France; 4 Oticon Medical – 2720 Chemin Saint Bernard, 06220 Vallauris, France

## Abstract

It is now well established that extensive musical training percolates to higher levels of cognition, such as speech processing. However, the lack of a precise technique to investigate the specific listening strategy involved in speech comprehension has made it difficult to determine how musicians’ higher performance in non-speech tasks contributes to their enhanced speech comprehension. The recently developed Auditory Classification Image approach reveals the precise time-frequency regions used by participants when performing phonemic categorizations in noise. Here we used this technique on 19 non-musicians and 19 professional musicians. We found that both groups used very similar listening strategies, but the musicians relied more heavily on the two main acoustic cues, at the first formant onset and at the onsets of the second and third formants onsets. Additionally, they responded more consistently to stimuli. These observations provide a direct visualization of auditory plasticity resulting from extensive musical training and shed light on the level of functional transfer between auditory processing and speech perception.

Acoustically, music and speech are two broad classes of sounds that share a number of characteristics. Both consist of quasi-periodic segments of rich harmonic complexity (notes and syllables) separated by silence, bursts of noise and/or transients and are organized with precise timing. These perceptual elements are mostly defined in terms of their spectral envelope, duration, and fundamental frequency^1^. The spectral envelope is one main component of a musical sound’s timbre (which allows identification of an instrument being played), whereas in speech its maxima, also called formants, are critical acoustic cues used to discriminate one vowel from another. In speech, as in music, pitch and rhythm are the two constituents of melody or prosody. Furthermore, both notes and vowels are perceived categorically, leading to a discretization of pitch space or formant space, respectively^2^. Finally, in real-world listening, sounds are rarely produced in isolation; however, the perception system demonstrates a remarkable ability to extract one auditory object from a background (e.g., the voice of your conversation partner in the presence of other speakers or one instrument in the orchestra)^3^. All of these commonalties suggest that similar basic auditory processes are involved during the analysis of both speech^4^ and music^5^.

Musical practice provides intensive training of these auditory capacities^6^. Musicians typically spend hours on tasks requiring the precise discrimination of sounds and melodies, leading to true auditory expertise. The most striking aspect of this sensory learning is the structural brain changes induced by instrumental training; musicians show increased grey matter volume in cortical areas of critical importance for music performance (auditory cortex, motor cortex, and visuo-spatial cortex)^7^,^8^,^9^. Such cortical reorganization can be connected to changes in cognitive abilities. Indeed, musicians have been demonstrated to outperform non-musicians in a number of non-speech auditory tasks. It is now well established that compared with non-musicians, musicians have larger auditory working memory^10^, finer frequency^10^,^11^,^12^,^13^ and duration discrimination^14^, enhanced auditory attention^13^, facilitated pitch processing^15^,^16^, better detection of tones masked by noise^13^ and greater perceptual acuity of rapid spectro-temporal changes^17^. This behavioral evidence for the benefit of musical experience on basic auditory skills has been supplemented with a series of electrophysiological studies. By directly measuring electromagnetic brain activity in response to various musical stimuli, researchers have been able to demonstrate increased sensitivity of musicians, compared with non-musicians, to mistuning^18^,^19^,^20^ and spectral complexity^21^,^22^.

The affinity between speech and music, described in the first paragraph, raises the question whether the improvement of basic auditory processes observed in musicians is domain-specific or can generalize beyond music processing to speech comprehension. There is general agreement that acuity enhancements observed in musical tasks percolate to higher-level skills. Musicians’ expertise is associated with a wide number of cognitive benefits for processing speech sounds, including word segmentation^23^, pitch processing in prosody^15^,^16^, and integration of metric structure^24^. Furthermore, a large literature has analyzed speech auditory brainstem responses (sABRs)^25^ in non-musicians and musicians, establishing that the latter show more robust encoding of speech sounds in the brainstem, even in situations of reverberation^26^ or noisy background^27^,^28^. Musicians also exhibit an enhanced brainstem representation of pitch contours^29^,^30^,^31^ and harmonics^27^,^32^,^33^. This superior encoding is significantly correlated with the amount of musical training^29^,^31^,^32^ and occurs from an early age^34^.

Taken together, these findings suggest that brain plasticity induced by musical expertise is not selective to musical stimuli but rather provides an advantage across domains. Notably, the effects of musical expertise transfer to speech processing and result in reinforced auditory skills and speech representations, leading to facilitated speech-in-noise perception. However, how musicians process their superior representations of speech to lead to better comprehension in noisy conditions remains unknown. In particular musical training could lead to a selective subcortical enhancement of relevant properties of speech sounds and eventually to a more focused auditory processing. Alternatively, musicians could exploit more efficient strategies involving different acoustic information than non-musicians.

In the present article, we asked whether musicians and non-musicians use the same acoustic cues during phoneme categorization in noise. This question was addressed here using a new auditory psychophysical method developed by some of the authors, which was named “Auditory Classification Image”.

The Auditory Classification Image (ACI) is a powerful tool and originates from the work of Ahumada on tone-in-noise detection^35^,^36^. Informally, ACIs can be thought of as a visual description of the participant’s listening strategy in an auditory categorization task. The experimental paradigm consists of introducing random fluctuations to the stimulus and then measuring the influence of these fluctuations on the participant’s behavior. This approach reveals how noise masking of different “parts” of the sound biases the listener toward a specific response. Several statistical methods have been proposed for the derivation of Classification Images from visual categorization experiments, including linear regression^35^,^36^, reverse correlation^37^,^38^, and generalized linear models (GLMs)^39^,^40^,^41^. The most recent developments in the field involve penalized GLMs (also called generalized additive models)^39^,^42^,^43^. This last technique offers sufficient power to translate Classification Images back to the auditory domain. This step has been performed in our group through the example of /aba/-/ada/ categorization, yielding a map of the stimulus regions in which noise consistently affects participants’ responses^44^. ACIs are a behavioral counterpart of spectro-temporal receptive fields (STRFs), a widely used model to capture the relationship between the acoustic characteristics of a stimulus and the firing of a specific auditory neuron^45^,^46^. At the population level, it has been demonstrated that the ACIs of several participants can be combined and analyzed using specific statistical tests from functional neuroimaging. This approach has been employed to unmask the auditory primitives used by normal-hearing participants to differentiate the syllables /da/ and /ga/47. In addition to the expected acoustic cues present in the F2 and F3 onsets, which have been previously determined through other methods, the authors were able to reveal a neglected source of information about the identity of the stimulus: the F1 onset.

The objectives pursued in the present study are twofold: 1) Determining whether musicians and non-musicians use different sets of acoustic cues when performing a /da/-/ga/ categorization in noise. 2) Estimating the specificity of each participant’s listening strategy, in both musicians and non-musicians. These issues translate into two testable hypotheses: (H1) Particularities of musicians’ listening strategies should be reflected in their ACIs. Therefore, there should be significant differences between musicians’ ACIs and ACIs estimated for a group of non-musician participants. (H2) A measure of specificity can be found by evaluating how the ACI from one participant successfully predicts the responses of other participants. Individual characteristics in the ACIs would result in low generalizability across participants.

## Methods

### Participants

Forty adults without any known audiological or neurological pathology participated in this study. They provided written informed consent and were paid 100€ for participation. They were divided into two groups: 20 musical experts, who reported more than 7 years of musical practice and started musical training before the age of 13, and 20 control participants (normal readers who reported no musical practice). Seventeen control participants were individuals already included in a previous study^47^. A pre-test confirmed that all participants had audiometric pure-tone thresholds ≤20 dB over the 125 Hz–8000 Hz range for both ears.

Two participants achieved unexpectedly low performances in the experiment, suggesting misunderstanding of task instructions, and their data were not included in further analyses. The two resulting groups (N = 2 × 19) were matched with regard to age, gender, and handedness (independent t-tests, all p > .05). Attentional capacities were evaluated using the Attention Network Test (ANT)^48^. Groups did not differ significantly (p > .05) in Alerting, Orienting and Conflict effect scores. Table 1 shows the mean characteristics of the participants in each group.

Musician participants were additionally administered questionnaires on their musical practice and were tested for absolute pitch using a simple pitch-naming task without feedback. The procedure was automatized with notes played on a synthetic piano, randomly drawn from the full-range of the keyboard (from C1 to B6). Eight participants obtained a score of at least 17/20 on this task, and will be further considered as absolute pitch possessors. All results are reported in Table 2.

The study was approved by the Comité d'évaluation éthique de l’Inserm/Institutional Review Board (IORG0003254, FWA00005831, IRB00003888). All methods were carried out in accordance with the approved guidelines.

### Stimuli

**Figure 1:**
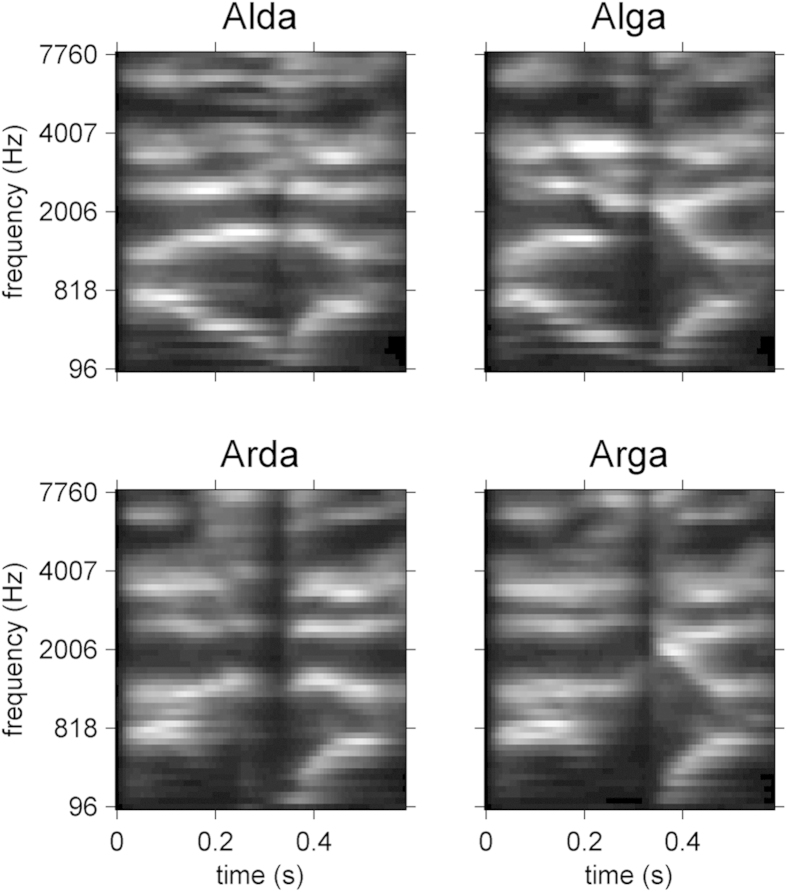
Cochleagrams of the four stimuli involved in the experiment. Parameters for spectral and temporal resolution are the same as those used for the derivation of ACIs (see details in the text).

The targets were 4 productions of VCCV non-words (/alda/, /alga/, /a
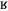
da/, and /a
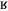
ga/) used in a previous behavioral study^47^. They were recorded by a male speaker in a soundproof booth (48 kHz; 16 bits). Inter-syllable gap durations were reduced such that the time onsets of the 2^nd^ syllable in all target sounds were the same (t = 328 ms), and they were then made equivalent with respect to duration (680 ms). The target sounds can be downloaded as .wav files at https://zenodo.org/record/12300/, and their cochleograms are shown in Fig. 1. For each participant, a set of 10,000 white-noise stimuli of the same length as the targets were generated and stored prior to the experiment. They can be found at the addresses https://zenodo.org/record/19104 and https://zenodo.org/record/23064.

On trial i, the stimulus presented consisted of one target signal, 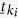, that was embedded in noise, 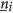 (both root-mean-square normalized), with a given *SNR*_*i*_, as shown in equation (1).
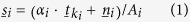


where 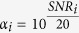 and 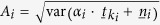, a factor allowing the power normalization of the stimulus. During presentation, all stimuli were preceded with a short Gaussian fade-in of Gaussian noise for the listener’s comfort.

### Experimental procedure

The stimuli were presented diotically over Sennheiser’s HD 448 headphones at each listener’s most comfortable sound pressure level. The participants were instructed to listen carefully to the speech sound and to press one of the two response keys as rapidly as possible according to whether they heard the last syllable as /da/ or /ga/. The response to trial *i* was denoted *r*_*i*_ = 0 for ‘da’ and 1 for ‘ga’). Repetition of the stimulus was allowed as many times as required, but this option was rarely used. In case the speech could not be identified, the subject was instructed to guess one of the two syllables.

Stimulus presentation was randomized across the two targets and was divided into 20 sessions of 500 trials each (10,000 trials total). To prevent excessive fatigue, the experiment was scheduled over 4 days. Typically, one day of the experiment lasted 2 h and consisted of a short practice block (~5 min), 3 sessions (~45 min) followed by a long break (~15 min), one cognitive test (~10 min), and then 3 more sessions of the main experiment (~45 min). Sessions were separated by 3 minute breaks. During the practice block, participants received automatic feedback about the accuracy of their answers, and the SNR was set to −11 dB. During the main experiment, however, no feedback was provided, and the SNR was adapted from trial to trial using a 3-down, 1-up staircase procedure^49^. The SNR was increased by one step after each incorrect response and decreased by one step after three consecutive correct responses from the last change in stimulus intensity. At the beginning of each session, the step size was set to 2 dB to accelerate convergence and then decreased by 10% each step until a step size of 0.2 dB was attained. The initial SNR level was −11 dB, and each session began with the final SNR of the previous session. This procedure, which was designed to target a threshold corresponding to 79% correct answers, allowed us to control for task difficulty over the course of the experiment even in the case of a momentary loss of attention. Data from all participants can be downloaded at the following addresses: https://zenodo.org/record/21808 and https://zenodo.org/record/19134.

### Derivation of Auditory Classification Images

ACIs were measured using the penalized GLM method^44^,^47^. Noisy stimuli 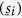 were pre-processed before entering the model, using Lyon’s cochlea model (quality factor = 8; overlapping = 40%)^50^. These cochleogram representations, binned at 15.6 ms in time and in 54 logarithmically spaced spectral bins between 96 and 7760 Hz, were used as predictors of the participant’s response. The estimation process was the same as in a previous study^47^, except for one notable improvement described below.

In the previous model, the prediction of the participant’s response was based on the cochleogram of the noise plus a two-level factor corresponding to the presented target. This dissociation between the noise and the target, inspired by the literature on visual Classification Images^39^,^43^, presupposes that these two elements are linearly combined in predictor space. However, this is not the case here because the cochleogram is not a linear operator. A more proper way to formulate the model is to directly use the cochleogram of the stimulus as the predictor (hereafter the cochleogram of stimulus 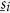 will be denoted 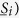. In line with the linear observer model derived from signal detection theory^42^,^51^, a decision variable is formed by taking the dot product of 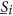 and an internal template 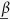 , the ACI, and adding the single parameter *c*, which reflects the general bias of the participant in favoring ‘da’ or ‘ga’:
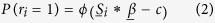


*ϕ* is a link function (here, an inverse logit function) that transforms the decision variable 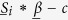 into an answer probability, 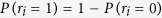. The model parameters 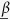 and *c* are estimated via the penalized likelihood (maximum a posteriori) of the GLM. The penalty chosen here is a smoothness prior and provides data-based, low-pass filtering of the ACI. The amount of penalty to be applied was determined by minimizing the mean cross-validated deviance of the participants’ models. The same level of penalty was obtained in both groups (musicians and non-musicians), thereby confirming a posteriori our choice of prior, and this value was applied to all estimations in this study.

This new method for the calculation of ACIs poses a problem, however, as it is sensitive to imbalances between correctly and incorrectly categorized trials, potentially leading to distorted representations of the underlying categorization template. Yet, the staircase algorithm necessitates that all participants obtain a 79% correct score. A simple solution adopted here was to balance the number of errors and correct categorizations before estimating the ACI. This goal was achieved by discarding a number of randomly chosen correct trials.

A comparison of the two methods using the same group of data (not shown) indicates that the ACIs obtained using this new method are less noisy and that the secondary cues are more distinct, although the estimation was based on ~4,200 trials versus 10,000 trials for the previous method.

### Statistical analysis

Once ACIs were estimated for all participants, they were individually z-scored and averaged to compute group ACIs, in accordance with previous work^52^. Because they are only approximations of the actual template used by the participant, encompassing a certain amount of estimation noise, it is imperative to apply some sort of statistical test to determine whether the resulting mean ACI is meaningful or can simply be due to a random process. Here, we used two different statistical tests from neuroimaging to decide which spectro-temporal regions truly reflected the listening strategy of a group of participants (t-test against zero with FDR correction^53^) or were significantly different between two groups of participants (t-test with cluster-based correction^54^).

## Results

Overall, participants in both groups obtained very similar patterns of correct response rates across all 4 signals (all p > .4). Their mean correct response rates were all nearly equal to 79% throughout the experiment thanks to the staircase algorithm (musicians: 79.28% ± 0.34% S.D.; non-musicians: 78.83% ± 0.40% S.D.). These percentages, although very close, were significantly different (t(36) = 3.6; p < .001). We estimated the sensitivity and decision criteria, as defined in signal detection theory^51^, for the two groups. Only sensitivity differed significantly (musicians: 1.68 ± 0.05 S.D.; non-musicians: 1.64 ± 0.04 S.D.; t(36) = 3.11; p = 0.0036). As a group, musician participants enrolled in this study also obtained better performances than the non-musician group in terms of SNR. Specifically, the mean SNR over all trials was significantly lower for musicians than for non-musicians (musicians: −13.37 dB ± 1.19 dB S.D.; non-musicians: −11.91 dB ± 1.01 dB S.D.; t(36) = −3.97; p < .001) resulting in a slightly superior recognition rate. The mean response time (RT) did not differ significantly between the groups (musicians: 1.37 s ± 0.18 s S.D.; non-musicians: 1.27 s ± 0.12 s S.D.; t(36) = 1.77; p = 0.086). These results are summarized in Table 1.

**Figure 2:**
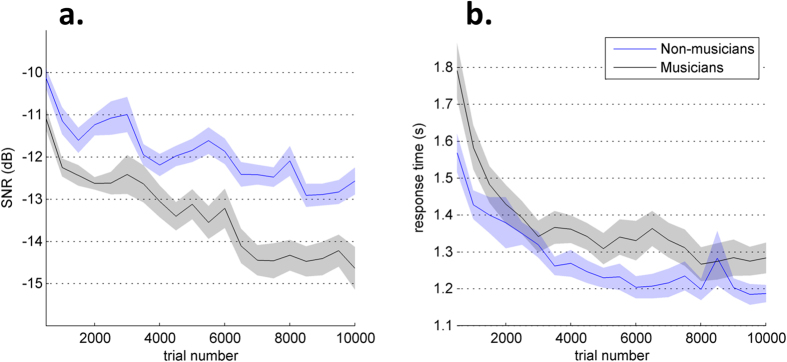
Evolution of SNR (a) and response time (b) over the course of the experiment, averaged across participants (the shaded region shows the s.e.m.). Each data point corresponds to a mean over 500 trials.

However, the participants’ performance level is not a stable state. Rather, SNR and RT decrease slowly over the course of the experiment (Fig. 2). A 2-way repeated-measures ANOVA showed significant effects of session number (F(19,36) = 26.93, p < .0001) and group (F(1,36) = 15.77, p = 0.0003) on SNR, with a significant interaction effect (F(19,684) = 1.83, p = 0.017). Thus, musicians were not only better performers in general but they also demonstrate faster learning. RTs were only affected by session number (F(19,36) = 32.88, p < .0001), and by a significant interaction between group and session (F(19,684) = 1.7, p = 0.0319).

Because musicians performed the task in significantly higher levels of background noise than non-musicians, SNR acts as a confounding variable in the comparisons between the two groups. Therefore, we additionally compared two subgroups of equivalent SNRs. For this purpose, we excluded 4 participants with mean SNRs that deviated more than 1.65 SD from the mean SNR of the non-musician group (e.g., outside the range of −11.91 dB ± 1.67 dB). Four additional non-musician participants were randomly discarded to result in two subgroups (N = 13) of equivalent SNR. Response times were also comparable (unpaired t-test, t(24) = −0.64, p = 0.53). Note, however, that the higher-performing musicians were obviously not included in the subgroup comparison.

**Figure 3:**
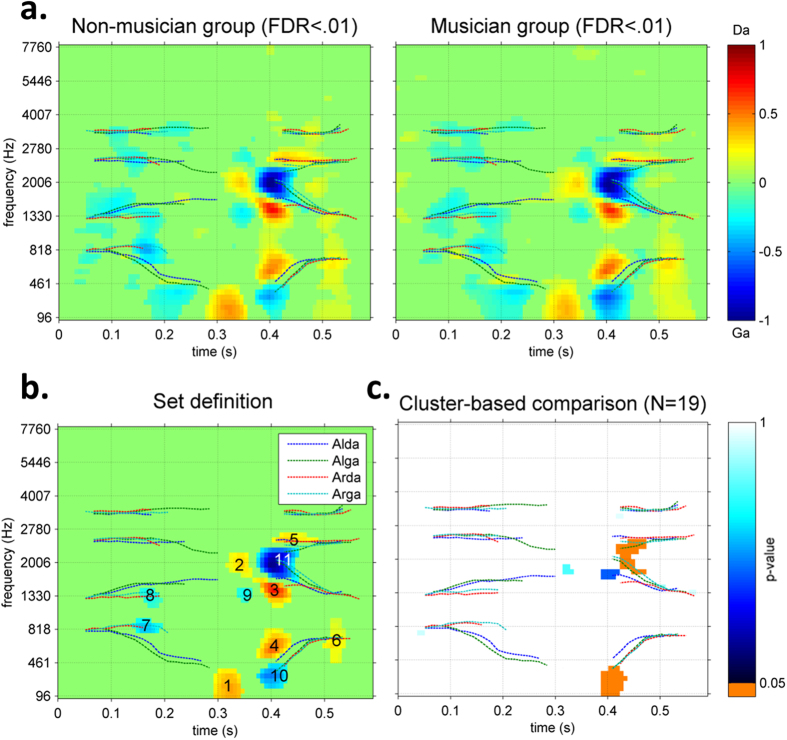
(**a**) ACIs for the two groups of participants (N = 19). Non significant weights (FDR > 0.01) are set to zero (**b**). Mean ACI over all 38 participants. Only weights sets (min. 7 adjacent weights with p < 10^−10^) are shown (**c**). Cluster-based nonparametric test between ACIs for the Non-musician and Musician groups (N = 19).

Individual ACIs were derived, z-scored, and group-averaged. The group-ACIs obtained are shown in Fig. 3a,b, with non-significant parameters set to zero (FDR > .01). High positive (red) and negative (blue) clusters of weights are time-frequency regions where noise biased the response of the participants towards ‘da’ or ‘ga’, respectively. ACIs for both groups demonstrated a very similar distribution of positive and negative weights.

For greater clarity, we defined 11 sets of weights by gathering CIs from all 38 participants regardless of their group, performing a t-test against zero for each weight, and considering only regions that exceeded the arbitrary threshold of p < 10^−10^ and formed a cluster of at least 7 adjacent significant pixels. Based on this profile, we characterized each set of weights by its size, time-frequency position (defined as the location of its centroid), spatial and temporal extent, sign (whether it was composed of positive or negative weights), and match with the acoustic features of the signals. The contours of all sets are plotted in Fig. 3c, and a summary of their characteristics is provided in Table 3.

As in STRFs, the ACI combines excitatory and inhibitory regions at close spectro-temporal positions to encode the presence of acoustic cues. Figure 3a,b. show that the main acoustic cue used by participants performing the task was located at the onsets of the F2 and F3 formant transitions in the second syllable (approximately 413 ms and 1960 Hz). The main acoustic cue consists of an inhibitory center in favor of the response ‘ga’ (set #11), with an excitatory surround in favor of the response ‘da’ (sets #2, #3 and #5). That is, the probability of the ‘ga’ answer increased in response to an increment in energy in set #11 and in response to a decrement in energy before, above and below this area. A similar configuration was observed at the onset of the F1 formant transition (sets #1, #4 and #10). Additionally, a number of weaker but significant weights were located in the first syllable and at the end of the second syllable (sets #6, #7 and #8).

A cluster-based nonparametric test indicated that musician listeners placed greater weight on the onsets of F2 and F3 (the latest part of set #11, p = 0.029) and on the onset of F1 (set #10, p = 0.027) than non-musician listeners (Fig. 3c). Arguably, differences in cue weighting between the two groups could simply be due to the global difference between the SNR at which they carried out the task (−13.37 dB on average for musicians versus −11.91 dB for non-musicians). To rule out this possibility, we also ran the same cluster-based test on two subgroups (N = 13) with equivalent SNRs. The same significant clusters were found (set #11: p = 0.048; set #10: p = 0.01), indicating that SNR differences were not the only cause of weighting changes between the musicians and non-musicians. We also made sure that these conclusions did not depend on the threshold used to delimit the clusters in the nonparametric test by replicating the above results with various thresholds values (α_threshold_ = .01, .025 and .05).

One advantage of the cluster-based analysis is that it makes no assumptions about the location of the effect. However, to obtain a more complete picture of the strategies of the two groups, we also performed a more classic ROI analysis on the mean weights in the previously defined sets. Table 3 shows the p-values for these t-tests. The hierarchy of the weights was the same in musicians and non-musicians, with relatively low inter-subject variability. Only sets #10 and #11 were weighted differently by the two groups, confirming the above results.

Similarly, we also investigated the specificity of each participant’s listening strategy for both groups. A straightforward way of evaluating this characteristic is to test how a model trained on one participant’s data can generalize to predict the responses of other participants. All goodness-of-fit measures are presented in terms of deviance (prediction error rates are also displayed for better understanding). As above, the proportions of correctly and incorrectly categorized trials were equated to make sure that the performance level of the participant did not impact the prediction of the model.

Ten-fold cross-validated predictions were calculated for each listener and termed “auto predictions”. This method provides a measure of how a model trained on a subset of the listener’s data can successfully predict other responses from the same participant. The data were randomly divided into 10 equal subsets, 9 of which constituted the training-set for fitting the GLM. Then, the predictive power of the resulting model was evaluated on the remaining subset (test-set). This operation was repeated 9 times until all subsets were used as test-sets once. Finally, the 10 deviance values were averaged to obtain the auto-prediction deviance for the participant. Auto-predictions were significantly better (unpaired t-test, t(36) = 2.7, p = 0.01) in the Musician group (233.41 ± 23.5 S.D.) than in the Non-Musician group (252.22 ± 19.1 S.D.).

**Figure 4:**
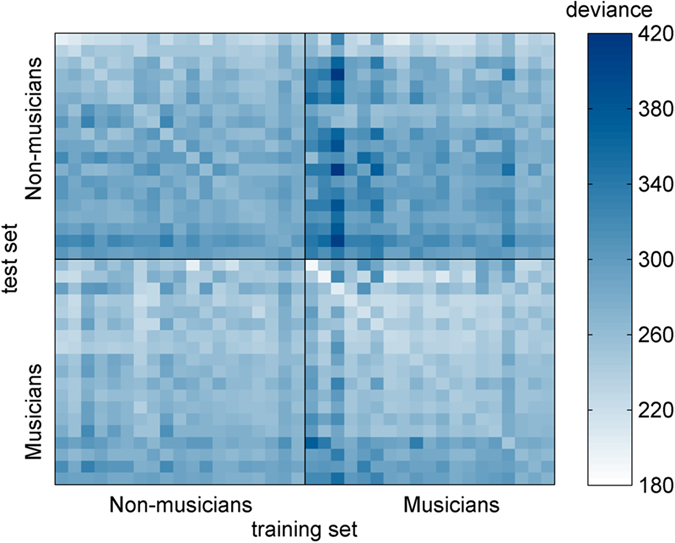
Cross-prediction deviances for all participants. Auto-predictions are represented along the diagonal of the matrix. Participants in each group are sorted by auto-prediction values.

Our aim was to compare the auto-prediction for each listener with his/her “cross-predictions”. The latter were calculated in precisely the same way as the auto-predictions except that the test-set used was taken from a different subject than the training-set. The matrix of cross-prediction values is shown in Fig. 4. As expected, a given model was better able to predict unseen data from the participant on which it had been trained than data from another participant (paired t-test, t(37) = −5.9805, p < 10^−6^). On average, the auto-prediction performance reached 69.1% correct predictions, whereas the mean cross-prediction performance was approximately 63.9% correct (both performances were superior to the chance level of 50%). As described in the Methods section, the amount of the penalty applied to all maximum a posteriori estimations calculated in this article was determined by minimizing the mean auto-prediction deviance across all participants. To confirm the accuracy of our approach, we also verified that the same value was obtained by minimizing the mean cross-prediction deviance instead.

Additionally, cross-prediction deviances within the Musician group were lower than those within the Non-Musician group (unpaired t-test, t(36) = −4.28, p < 10^−3^). It could not be inferred from the prediction data that GLMs fit on the musicians’ responses were better predictors of the whole group of 38 participants than GLMs fit on the non-musicians’ responses (unpaired t-test, t(36) = −1.46, p = 0.15). However, when averaging across training-sets instead of across test-sets, the musicians’ responses were more accurately predicted than the non-musicians’ responses (unpaired t-test, t(36) = −3.44, p = 0.0015).

A measure of the specificity of a listener’s strategy can be obtained by taking the difference between his/her auto-prediction deviance and the cross-prediction deviance of his/her data produced by GLMs fit to the other participants. This value is higher when the subject’s responses are better predicted by his/her own ACI than by others’ ACIs. The specificity measures did not differ significantly between the two groups (Non-Musicians: −31.76 ± 7.04 S.D.; Musicians: −30.71 ± 14.88 S.D., unpaired t-test, t(36) = −0.28, p = 0.78).

## Discussion

In this study, we aimed to determine how the enhanced cognitive abilities of expert musicians in non-speech acoustic tasks, compared with those of non-musicians, are linked to their better performance in speech-in-noise comprehension. For this purpose, we used the Auditory Classification Image approach in a simple phoneme categorization task in noise. Nineteen musicians and 19 normal-hearing participants with no musical practice were asked to discriminate the final syllable in 4 non-word stimuli (/alda/, /alga/, /a
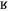
da/, and /a
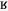
ga/). The noise level was adjusted throughout the experiment so that the percentage of correct answers was 79%. ACIs were derived for each participant to reveal the precise time-frequency regions where noise influenced his phonemic categorization.

As expected, musicians enrolled in this study demonstrated better phoneme perception in background noise than non-musicians. Although the percentages of correct answers were close to 79% in both groups, the musicians performed the task at an average SNR that was 2 dB less favorable. Furthermore, their estimated sensitivities were slightly, but significantly, superior to those of non-musicians. These results confirm that musical expertise enhances resistance to noise, as has been demonstrated in previous studies^10^. This validates our experimental paradigm and enables us to explore the ACIs to identify correlates of this behavioral effect in the acoustic cues used. A tentative explanation for the slight, marginally significant, difference in response times between musicians and non-musicians could be that musicians are more meticulous in the task, being slower in their responses but obtaining better performances. Additionally the two groups show different evolution of performances with time, as indicated by the significant interaction effects between trial number and SNR or response time, suggesting faster learning for musicians.

All individual ACIs exhibited similar weight patterns, consistent with the results of a previous study that used a slightly different model (see Methods section) and a smaller number of participants^47^. The main sets of weights are depicted in Fig. 3c, and a summary of their characteristics is provided in Table 3. At least two acoustic cues appeared to be involved in the /da/-/ga/ categorization: 1) the configuration of the F2/F3 transitions was different for the two syllables (close onsets for /ga/, distant onsets for /da/). This property was encoded by weights in sets #3, #5 and #11. 2) The position of F1, although less informative for proper identification of the consonant, was precisely reflected in the ACI by sets #4 and #10. Both cues were preceded by opposite weights in the same frequency bands (sets #1, #2 and #9), indicating that energy detection in one channel at time t also depends on energy in the same channel at previous instances in time. Although the general listening strategies used by the participants in this study appear to have been very similar, we cannot exclude that there may have been fine group differences.

The main goal of this study was to determine whether and how the extensive training of auditory abilities in music experts modifies the auditory cues used for the categorization of /da/ and /ga/ in noise. If this was the case, there should have been a significant difference between the ACIs of the Musician and Non-Musician groups. Indeed, a cluster-based, nonparametric test indicated that musicians placed greater weights on the end of the central negative set #11 (p = 0.029) (Fig. 3c). Therefore, the contour of the auditory cue more closely traced the second and third formant transitions in the second syllable. Another difference was found for the second acoustic cue, more precisely in set #10. These two acoustic cues were more heavily weighted by musician participants, suggesting better selectivity for the task-relevant characteristics of the stimuli, which was enabled by the hypersensitivity of their over-trained auditory skills.

This picture is complicated by a potentially confounding variable: differences between the ACIs of the two groups can be explained by the differences in performance between the two groups, reflected by the lower SNRs for musicians compared to non-musicians. This possibility has been ruled out, however, because the two subgroups that were matched in SNR (and of equivalent reaction times) showed the same pattern of differences in their ACIs. It is noteworthy that although the higher-performing musicians were not included in this last comparison, the result remains the same. This finding suggests that the plasticity of the auditory process induced by musical practice might impact speech processing even when it provides no or very little advantage for comprehension. To further investigate this effect we performed a ROI analysis on the ACIs of the low- and high- performing musicians. Significant differences were obtained only on sets #6 (t(17) = −2.2597, p = 0.037) and #7 (t(17) = 2.7183, p = 0.015) (the complete list can be found as Supplementary Table S1 online). This set of results would imply that all musician experts selectively focus on the relevant information contained in the speech stimuli, but some of them may be unable to process it effectively by combining it with secondary cues. However, the sample size being very small (N = 6 for the high-performer subgroup), further study is needed to confirm this tentative explanation.

Absolute pitch, a change in sensory representations of tones associated to early musical training for some people^55^, may pose a quite serious confound for the interpretability of the results. Indeed the possession of this ability might pave the way for alternative strategies in our task, or enforce the existing strategy, possibly explaining the differences between musicians’ and non-musicians’ ACIs. This concern needs to be addressed here by a complementary analysis. Fortunately, it was possible to evaluate the effect of absolute pitch by dividing the musicians group into 2 subgroups, according to whether or not they scored more than 17/20 on the pitch-naming task. The differences between the ACIs of musicians with (N = 8) or without (N = 11) absolute pitch was determined by means of a ROI analysis. None of the 11 t-tests indicated a significant differential weighting of the cues (all p > 0.05). The detailed results are reported in Supplementary Table S1 online. This result supports the claim that the change in auditory processing observed in musicians, compared to non-musicians, is driven by group differences in musical experience rather than by a subgroup of absolute pitch possessors.

It may seem contradictory that intensive musical training, which has been shown to confer sharper tuning of cochlear filter responses^56^ and finer spectral and temporal discrimination^10^,^11^,^12^,^13^,^14^, resulted in larger cues in the ACI in the current study. However, in the context of speech-in-noise perception, this adaptation provides an advantage because it is a way to obtain more reliable estimates of formant positions by gathering information from a wider area. To summarize, although they used the same listening strategy as non-musicians, musicians selectively focused on a small time-frequency region critical for correct /da/-/ga/ categorization. In light of these results, the musicians’ performance could be at least partially explained by the enhanced selectivity for the most behaviorally relevant aspects of the sound. One tentative explanation is that the more robust encoding of speech in the musicians’ brainstem and their enhanced auditory abilities pave the way for more flexible cue weighting, such as the one described here. Even if they use the same acoustic cues, their extensive training of auditory functions makes them better able to focus on those cues and to not be influenced by non-behaviorally relevant acoustic information.

Another aspect that we wanted to investigate in this study was the specificity of each participant’s listening strategy. This was performed by comparing the auto-predictions (prediction of a subject’s response using his own ACI) and the cross-predictions (prediction of a subject’s responses using the ACIs of the other subjects) (Fig. 4). We first confirmed the validity of this approach by verifying that the ACIs were generalizable from one participant to another: on average, the cross-prediction rate was significantly above chance (64%), although it was inferior to the mean auto-prediction rate (69%). While a study using Bubble Images, a method closely related to ACIs, demonstrated that intelligibility maps can be used to predict the intelligibility of other phonemes and/or other talkers^57^, the current result shows that a comparable generalization is possible across listeners. The comparison of cross-prediction deviances between the two groups offers another insight into the differences in speech processing induced by musical training. On average, musicians’ data were more accurately cross-predicted than non-musicians’ responses (p = 0.0015), indicating that they were more consistent in their responses. This result could be due to a reduction of internal noise in the Musician group (fewer unpredictable mistakes), due perhaps to their increased sustained auditory attention capabilities^13^. However, musicians and non-musicians did not seem to differ in terms of the specificity of their ACIs, based on the gap between auto-prediction and mean cross-prediction for each participant in both groups (p = 0.78).

Of course, the acoustic cues identified depend on the categorization task studied (/da/-/ga/ categorization), and their precise time-frequency locations depend on the particular utterances presented. Nonetheless, our results demonstrate a phenomenon that is highly unlikely to be limited to this particular choice of phonemes and utterances. Furthermore, due to the high degree of nonlinearity in the speech comprehension process, it is possible that the exact weight ratio in the ACIs do not exactly reflect the real relative importance of acoustic cues^58^. However, this does not invalidate the difference in weighting between the two groups. Because the ACIs are estimated using the same set of targets, we completely avoid the issue of stimulus-dependent estimation.

A recent series of studies have questioned the initial finding of Parbery-Clark and colleagues^10^ that musicians have a general enhancement of speech-in-noise comprehension compared to non-musicians. They varied the type of task used, the targets, and the complexity of the maskers, showing that generally, the group difference was very small and in most cases non-significant^59^,^60^,^61^. They presented a plausible explanation for this absence of positive results: the musicianship advantage would only be revealed in the most difficult listening situations^59^. However, this interpretation is challenged by our observations. Indeed, a significant difference was obtained in our study using a forced-choice task with word targets, which is less demanding than the task used by Boebinger *et al.* (open-choice task with sentence targets). Furthermore, our adaptive staircase algorithm targeted a higher percentage of correct responses (79% compared with 50% in the previous study). Two alternative explanations can be proposed. First, the stronger effect of musicianship in our study could be due to a more strict selection of participants compared to the previous studies: non-musicians had no practice of an instrument, even for less than 2 years^10^,^59^,^60^,^61^. Our subjects were also required to have pure-tone thresholds better than 20 dB at the audiometric test, whereas normal hearing was not tested in Boebinger *et al.*^59^. These two factors can be a major source of inter-individual variability: on one hand, even as little as 6 months of musical training is sufficient to influence linguistic abilities^62^; on the other hand, musicians are more likely to experience hearing problems. Another explanation could be related to the non-naturalness of the forced-choice task we used. Here, we hypothesized that improved frequency discrimination enhances formant trajectory detection, thus resulting in better /da/-/ga/ categorization in noise. Nonetheless, it is possible that this ability does not strongly impact speech-in-noise perception in more natural listening situations, such as those employed in the studies above^59^,^60^,^61^.

In this paper, we showed that the ACI is a suitable tool for providing direct visualization of auditory plasticity resulting from intensive musical training. We also showed its effect on speech perception. This approach fills a gap in the current debate on the beneficial effects of musical training on speech perception. In line with what has been shown previously in sABR studies^27^,^29^,^30^,^31^,^32^,^33^ and in psychoacoustic studies^10^,^11^,^12^,^13^,^14^,^15^,^16^,^17^, we were able to demonstrate an enhancement of specific acoustic characteristics in the encoding/decoding of speech sounds. One important aspect of our method, however, is that it is based on speech comprehension data instead of on electrophysiological data or behavioral data for non-speech sounds. Hence, the acoustic cues identified are those that are behaviorally relevant for the phoneme categorization task (whereas sABR representations, for example, show all of the information extracted from the signal, even the information that is not used in later stages of the comprehension process). In the context of this study, the ACI approach allowed us to determine which cues were actually - not only potentially - better extracted and combined by musicians to yield better speech-in-noise perception. As such, this method provides a bridge between two sets of evidence: the existence of a musicianship advantage in speech-in-noise perception on one hand and improvements of basic auditory abilities on the other hand. This article has set the stage for future studies using the ACI approach to investigate group differences in auditory plasticity. In contrast with the enhancement of speech perception, another interesting application would be to explore its impairments, such as in dyslexia.

## Additional Information

**How to cite this article**: Varnet, L. *et al.* How musical expertise shapes speech perception: evidence from auditory classification images. *Sci. Rep.* **5**, 14489; doi: 10.1038/srep14489 (2015).

## Supplementary Material

Supplementary Information

## Figures and Tables

**Table 1:** Summary of the characteristics of the two groups.

Variable	Musicians	Non-musicians	t-test
Age (year)	23 (±2.89 S.D.)	22.68 (±4.39 S.D.)	p = 0.78
Gender (M/F)	9/5	6/13	
Handedness	64.21 (±53.99 S.D.)	73.95 (±57.72 S.D.)	p = 0.61
ANT			
Alerting effect	29.95 (±22.68 S.D.)	32.37 (±21.97 S.D.)	p = 0.78
Orienting effect	46.05 (±14.98 S.D.)	40.21 (±23.25 S.D.)	p = 0.36
Conflict effect	131.74 (±49.13 S.D.)	131.16 (±39.95 S.D.)	p = 0.97
Main experiment			
Score (%)	79.29 (±0.35 S.D.)	78.83 (±0.42 S.D.)	p = 0.0008*
SNR (dB)	−13.37 (±1.22 S.D.)	−11.91 (±1.04 S.D.)	p = 0.0004*
Reaction Time (s)	1.37 (±0.19 S.D.)	1.28 (±0.13 S.D.)	p = 0.11
Sensitivity d’	1.68 (±0.05 S.D.)	1.64 (±0.04 S.D.)	p = 0.0036*
Decision criterion	0.638 (±0.128 S.D.)	0.639 (±0.125 S.D.)	p = 0.97

**Table 2:** Details of all participants’ musical experience.

Musician	Age onset (year)	Years of practice	Instrument	Absolute pitch test (/20)
#1	13	7	Double bass; guitar	1
#2	6	20	Trombone	17
#3	6	13	Piano	20
#4	4	17	Violoncello	19
#5	6	15	Violin	8
#6	5	14	Accordion	17
#7	5	22	Violin; piano	18
#8	7	14	Violin	10
#9	5	18	Double bass	20
#10	6	16	Violin	6
#11	5	22	Piano	20
#12	7	13	Piano	13
#13	5	14	Flute; bassoon	2
#14	3.5	18	Opera singing	5
#15	5	21	violin, viola	15
#17	7	10	guitar	19
#18	7	13	guitar	2
#19	7	17	Double bass	14
#20	5	17	viola da gamba; bowed viol	8

**Table 3:** Summary of the characteristics of all sets of weights, sorted by bias and latency.

Set	size (pxls)	Centroid		Extent		Correspondence with formants	Bias towards	Set weights				
		t (ms)	f (Hz)	t (ms)	f (Hz)			NM (mean)	NM (SD)	M (mean)	M (SD)	t-test (M vs. NM)
#1	41	317,9	215,8	51,04	256,8	offset F1, 1st syllable	‘da’	0,0173	0,0097	0,0158	0,0065	p = 0,58
#2	25	340,8	1913	43,75	429,5	onset F2/F3, 2nd syllable	‘da’	0,0070	0,0030	0,0075	0,0026	p = 0,60
#3	34	406	1383	65,63	425,4	onset F2, 2nd syllable	‘da’	0,0166	0,0047	0,0160	0,0048	p = 0,68
#4	42	405	658,4	58,33	300,6	onset F1, 2nd syllable	‘da’	0,0158	0,0060	0,0171	0,0044	p = 0,45
#5	19	443,5	2548	80,21	137,7	onset F3, 2nd syllable	‘da’	0,0066	0,0018	0,0052	0,0022	p = 0,051
#6	34	521,6	697,1	36,46	489,7	offset F1, 2nd syllable	‘da’	0,0075	0,0050	0,0090	0,0025	p = 0,27
#7	18	166,1	876,4	51,04	133,5	offset F1, 1st syllable	‘ga’	−0,0067	0,0036	−0,0060	0,0030	p = 0,50
#8	17	173,9	1297	36,46	244,9	offset F1, 2nd syllable	‘ga’	−0,0040	0,0017	−0,0042	0,0020	p = 0,63
#9	10	350	1347	21,88	166,4	onset F2, 2nd syllable	‘ga’	−0,0029	0,0010	−0,0023	0,0017	p = 0,17
#10	32	404,9	301,3	51,04	209,2	onset F1, 2nd syllable	‘ga’	−0,0113	0,0066	−0,0180	0,0043	p = 7.10^−4^*
#11	60	413,9	1960	72,92	645,6	onset F2/F3, 2nd syllable	‘ga’	−0,0389	0,0076	−0,0461	0,0061	p = 2.10^−3^*
