# Supplementary material for: How musical expertise shapes speech perception: evidence from auditory classification images
Source: Sci Rep. 2015 Sep 24;5:14489. doi: 10.1038/srep14489 (PMC4585866; doi:10.1038/srep14489)
Supplement: Supplementary Information [file srep14489-s1.doc]

**Supplementary information**

**Title:** How musical expertise shapes speech perception: evidence from auditory classification images.

**Authors:** Léo VARNET^a,b,c*^, Tianyun WANG^a,b,c^, Chloé PETER^a,c^, Fanny MEUNIER^b,c^, Michel HOEN^a,c,d^

| **Set** | High-perform. (mean) | High- perform. (SD) | Low- perform. (mean) | Low- perform. (SD) | t-test (low- vs. high-perform.) | Absolute pitch (mean) | Absolute pitch (SD) | Non absolute pitch (mean) | Non absolute pitch (SD) | t-test (absolute pitch vs. non absolute pitch) |
| --- | --- | --- | --- | --- | --- | --- | --- | --- | --- | --- |
| **#1** | 0,0166 | 0.0023 | 0,0154 | 0.0078 | p=0,71 | 0,0166 | 0,0040 | 0,0152 | 0,0080 | p=0,66 |
| **#2** | 0,0080 | 0.0032 | 0,0072 | 0.0024 | p=0,58 | 0,0082 | 0,0030 | 0,0069 | 0,0023 | p=0,32 |
| **#3** | 0,0177 | 0.0031 | 0,0152 | 0.0053 | p=0,30 | 0,0151 | 0,0045 | 0,0166 | 0,0050 | p=0,51 |
| **#4** | 0,0195 | 0.0027 | 0,0160 | 0.0047 | p=0,11 | 0,0157 | 0,0056 | 0,0181 | 0,0033 | p=0,26 |
| **#5** | 0,0057 | 0.0014 | 0,0050 | 0.0026 | p=0,56 | 0,0051 | 0,0016 | 0,0053 | 0,0027 | p=0,81 |
| **#6** | 0,0108 | 0.0022 | 0,0082 | 0.0023 | **p=0,037*** | 0,0092 | 0,0032 | 0,0089 | 0,0021 | p=0,77 |
| **#7** | -0,0084 | 0.0028 | -0,0049 | 0.0025 | **p=0,015*** | -0,0069 | 0,0038 | -0,0054 | 0,0022 | p=0,31 |
| **#8** | -0,0045 | 0.0028 | -0,0041 | 0.0016 | p=0,68 | -0,0044 | 0,0025 | -0,0041 | 0,0016 | p=0,72 |
| **#9** | -0,0020 | 0.0020 | -0,0024 | 0.0016 | p=0,65 | -0,0020 | 0,0017 | -0,0025 | 0,0017 | p=0,56 |
| **#10** | -0,0167 | 0.0030 | -0,0185 | 0.0047 | p=0,40 | -0,0187 | 0,0041 | -0,0174 | 0,0045 | p=0,51 |
| **#11** | -0,0471 | 0.0087 | -0,0457 | 0.0048 | p=0,67 | -0,0469 | 0,0062 | -0,0456 | 0,0063 | p=0,67 |

Supplementary Table S1. Set weights and p-values for the ROI analyses between high- and low- performing musicians, and between musicians with and without absolute pitch.
